# Large airway T cells in adults with former bronchopulmonary dysplasia

**DOI:** 10.1186/s12931-024-02717-1

**Published:** 2024-02-09

**Authors:** Jing Gao, Petra Um-Bergström, Melvin Pourbazargan, Eva Berggren-Broström, ChuanXing Li, Heta Merikallio, Riitta Kaarteenaho, Nichole Stacey Reinke, Craig E Wheelock, Erik Melén, Lindén Anders, Åsa M Wheelock, Georgios Rassidakis, Cristian Ortiz-Villalon, Magnus Carl Sköld

**Affiliations:** 1 Respiratory Medicine Division, Department of Medicine Solna, Center for Molecular Medicine (CMM), Karolinska Institutet, Stockholm, 171 76 Sweden; 2 Department of Pediatrics, Sachs’ Children and Youth Hospital, Södersjukhuset, Stockholm, Sweden; 3 Department of Clinical Science and Education, Karolinska Institutet, Södersjukhuset, Stockholm, Sweden; 4 Department of Emergency and Reparative Medicine, Karolinska University Hospital, Stockholm, Sweden; 5 Research Unit of Internal Medicine and Medical Research Center Oulu, University of Oulu, Oulu University Hospital, Oulu, Finland; 6 Unit of Integrative Metabolomics, Institute of Environmental Medicine, Karolinska Institutet, Stockholm, Sweden; 7 Centre for Integrative Metabolomics and Computational Biology, School of Science, Edith Cowan University, Perth, Australia; 8 Department of Respiratory Medicine and Allergy, Karolinska University Hospital, Stockholm, Sweden; 9 Gunma University Initiative for Advanced Research (GIAR), Gunma University, Maebashi, Japan; 10 Division of Lung and Airway Research, Institute of Environmental Medicine, Karolinska Institutet, Stockholm, Sweden; 11 Department of Oncology and Pathology, Karolinska Institutet, Karolinska University Hospital, Stockholm, Sweden; 12 Department of Pathology, Karolinska University Hospital, Stockholm, Sweden

**Keywords:** Bronchopulmonary dysplasia, Asthma, Adults, Lymphocytes, Bronchial wash

## Abstract

**Background:**

Bronchopulmonary Dysplasia (BPD) in infants born prematurely is a risk factor for chronic airway obstruction later in life. The distribution of T cell subtypes in the large airways is largely unknown.

**Objective:**

To characterize cellular and T cell profiles in the large airways of young adults with a history of BPD.

**Methods:**

Forty-three young adults born prematurely (preterm (*n* = 20), BPD (*n* = 23)) and 45 full-term-born (asthma (*n* = 23), healthy (*n* = 22)) underwent lung function measurements, and bronchoscopy with large airway bronchial wash (BW). T-cells subsets in BW were analyzed by immunocytochemistry.

**Results:**

The proportions of both lymphocytes and CD8 + T cells in BW were significantly higher in BPD (median, 6.6%, and 78.0%) when compared with asthma (3.4% and 67.8%, *p* = 0.002 and *p* = 0.040) and healthy (3.8% and 40%, *p* < 0.001 and *p* < 0.001). In all adults born prematurely (preterm and BPD), lymphocyte proportion correlated negatively with forced vital capacity (*r*= -0.324, *p* = 0.036) and CD8 + T cells correlated with forced expiratory volume in one second, FEV_1_ (*r*=-0.448, *p* = 0.048). Correlation-based network analysis revealed that lung function cluster and BPD-birth cluster were associated with lymphocytes and/or CD4 + and CD8 + T cells. Multivariate regression analysis showed that lymphocyte proportions and BPD severity qualified as independent factors associated with FEV_1_.

**Conclusions:**

The increased cytotoxic T cells in the large airways in young adults with former BPD, suggest a similar T-cell subset pattern as in the small airways, resembling features of COPD. Our findings strengthen the hypothesis that mechanisms involving adaptive and innate immune responses are involved in the development of airway disease due to preterm birth.

**Supplementary Information:**

The online version contains supplementary material available at 10.1186/s12931-024-02717-1.

## Abstract (significance)

**What is already known on this topic:**

Bronchopulmonary Dysplasia (BPD) in infants born prematurely is a risk factor for chronic airway obstruction in adulthood. Cytotoxic T cells are increased in bronchoalveolar lavage in adults with former BPD.

**What this study adds:**

We are able to demonstrate that inflammatory cell profiles in the bronchial wash from the large airway of preterm adults differ from healthy subjects born at term. Subjects with a history of BPD display an increased proportion of lymphocytes, mainly CD8 + T cells, and a lower proportion of CD4 + T cells. Furthermore, the number of cytotoxic T cells is associated with lung function.

**How this study might affect research practice and/or policy:**

This study provides evidence of an immunological profile characterized by increased cytotoxic T cells in adults born prematurely, which suggests the involvement of both adaptive and innate immune mechanisms in the development of airway disease associated with preterm birth.

**Supplementary Information:**

The online version contains supplementary material available at 10.1186/s12931-024-02717-1.

## Introduction

Preterm birth causes abnormal lung development and is known to increase the risk of pulmonary complications including chronic airway obstruction later in life [1, 2]. Approximately 6–12% of all pregnancies in western countries ends prematurely [3, 4]. These infants may develop bronchopulmonary dysplasia (BPD), especially when birth weight is less than 1000 g [5–8]. BPD is a form of neonatal chronic lung disease [9], defined as the need for oxygen therapy up to the 28th day of life for children born before gestational age 36 weeks [10]. Importantly, BPD is associated with immature lung tissue affected by reparative processes, impaired alveolarization, and dysmorphic vascular growth [11, 12], while structural changes may also persist into adulthood [13, 14]. Chronic airway obstruction associated with preterm birth is expected to be a growing group in the future, because of an increasing number of survivors reaching adult life [15]. Although preterm birth is known to enhance the risk for airway disease later in life [16], the mechanisms are poorly understood.

Infants born prematurely are often regarded as having asthma and are frequently treated with asthma medications [17, 18]. In asthma, inflammation is present both in the large and small airways [19], whereas in chronic obstructive pulmonary disease (COPD), inflammation is found also in the lung parenchyma [20–23]. Lymphocytes are known to be involved in mechanisms behind airway obstruction [24]. Thus, type 2 CD4 + T cells are involved in asthma pathogenesis [25, 26], whilst the role of CD8 + T cells has been suggested to balance the responses of CD4 + T cells through the secretion of Interferon-γ IFN-γ [27]. In chronic bronchitis, CD8 + T-lymphocytes, neutrophils and CD68 + monocytes/macrophages predominate [21, 28]. In stable COPD, the inflammatory profile is characterized by an increased number of T-lymphocytes, particularly CD8 + T cells, macrophages, and neutrophils [29, 30]. In preterm infants, respiratory distress syndrome is associated with a lower T cell count and a higher proportion of activated cells [31].

In a paper by Galderisi et al. [32] it was reported thickened basement membrane and increased airway lymphocytes, predominantly CD8 + T cells, in adolescent survivors of BPD, underscoring the long-term histopathological impact of the disease. In addition, we have recently shown that adults with a history of BPD have an increased proportion of activated CD3 + CD8 + cells, a decrease in CD3 + CD4 + T cells and a reduced CD4/CD8 ratio in bronchoalveolar lavage (BAL) fluid [33]. Since BAL represents small airways and obstructive airway disease also involves the large airways, our aim was to characterize the cellular profile in large airways. To accomplish this, we preformed bronchial wash in adult individuals with previous BPD and compared them with prematurely born without BPD, allergic asthma and healthy controls.

## Method

### Participants

The participants were included in Lung Obstruction in Adulthood of Prematurely Born (LUNAPRE) study [18, 33, 34], registered at www.clinicaltrials.gov/ct/show/NCT02923648 (Study Registration Date/First Posted Date: October 4, 2016; Actual Study Start Date: March 1, 2013). The study includes adults (≥ 18 years of age) born preterm (< 32 weeks gestational age (GA) and full-term (> 37 weeks GA). The adults were categorized into four groups: preterm-born participants with a neonatal diagnosis of BPD [10] (BPD, *n* = 23), preterm-born participants without BPD (Preterm, *n* = 20), patients with mild allergic asthma (Asthma, *n* = 22) and healthy controls (Healthy, *n* = 23). BPD was defined as ≥ 28 days of needing supplemental oxygen for children born before 32 GA weeks, while severity was determined at 36 GA weeks [10, 33]. BPD severity was defined based on oxygen need at 36 week at postmenstrual age (PMA) or discharge, as mild (grade 1, need for breathing room air), moderate (grade 2, need for < 30% oxygen), and severe (grade 3, need for ≥ 30% oxygen and/or positive pressure) [10]. The preterm-born participants were recruited from a pre-existing cohort at Sachs’ Children and Youth Hospital in Stockholm, Sweden where they had been admitted between 1992 and 1998 [35]. The subjects were assessed in two Swedish hospitals in Stockholm (Karolinska University Hospital Solna and Sachs’ Children and Youth Hospital, Södersjukhuset) during the years 2013 to 2017. All patients with allergic asthma had a positive methacholine challenge test with a decrease in FEV_1_ ≥ 20% and presence of IgE sensitization to any airborne allergen [34], employing Phadiatop® (Thermo Fisher Scientific; Pharmacia, Uppsala, Sweden) which includes birch, timothy, mugwort, cat, dog, horse dander, mold (Cladosporium herbarum), and house dust mite (Dermatophagoides pteronyssinus) [34]. Analyses were done at the clinical laboratory of Karolinska University Hospital, Stockholm, Sweden. None of the participants received any ongoing anti-inflammatory treatment or had have respiratory tract infections for ≥ 3 months prior to inclusion. All participants were never smokers and they all provided written informed consent and the study was approved by the regional ethics committee in Stockholm [33] (ref: 201211872-31/4, Ethics Date for the Trial: November 21, 2012). In a previous study from the same cohort [33], 90 subjects were included for collection of BAL fluid. In the present study, one patient from the BPD group provided BW, but BAL were not collected due to clinical constraints. Therefore, the study includes 23 subjects in the BPD group. We encountered challenges in obtaining large airway specimens from one healthy individual and two adults with a preterm history.

### Data from the perinatal and postnatal period

Information on perinatal and neonatal history was collected from the Swedish Medical Birth Registry and medical charts, including the information on treatment with prenatal steroids and corticosteroids used in postnatal period, Apgar-score (APGAR), GA at birth, birth weight, instillation of surfactant, number of days on a ventilator, Continuous Positive Airway Pressure (CPAP) and supplemental oxygen [10].

### Lung function

Dynamic spirometry and standardized procedures were performed according to the recommendations of the American Thoracic Society/European Respiratory Society guidelines [33, 36, 37], using the Sensormedics 6200 body plethysmograph (SensorMedics; Yorba Linda, CA, USA). The values of forced vital capacity (FVC), forced expiratory flow in 1 s (FEV_1_), fractional exhaled nitric oxide (FeNO), diffusing capacity of the lung for carbon monoxide (DLCO), and residual volume (RV), total lung capacity (TLC) ratio, and methacholine challenge test) were collected [33].

### Bronchoscopy and bronchial wash specimens from the large airways

**Fig. 1:**
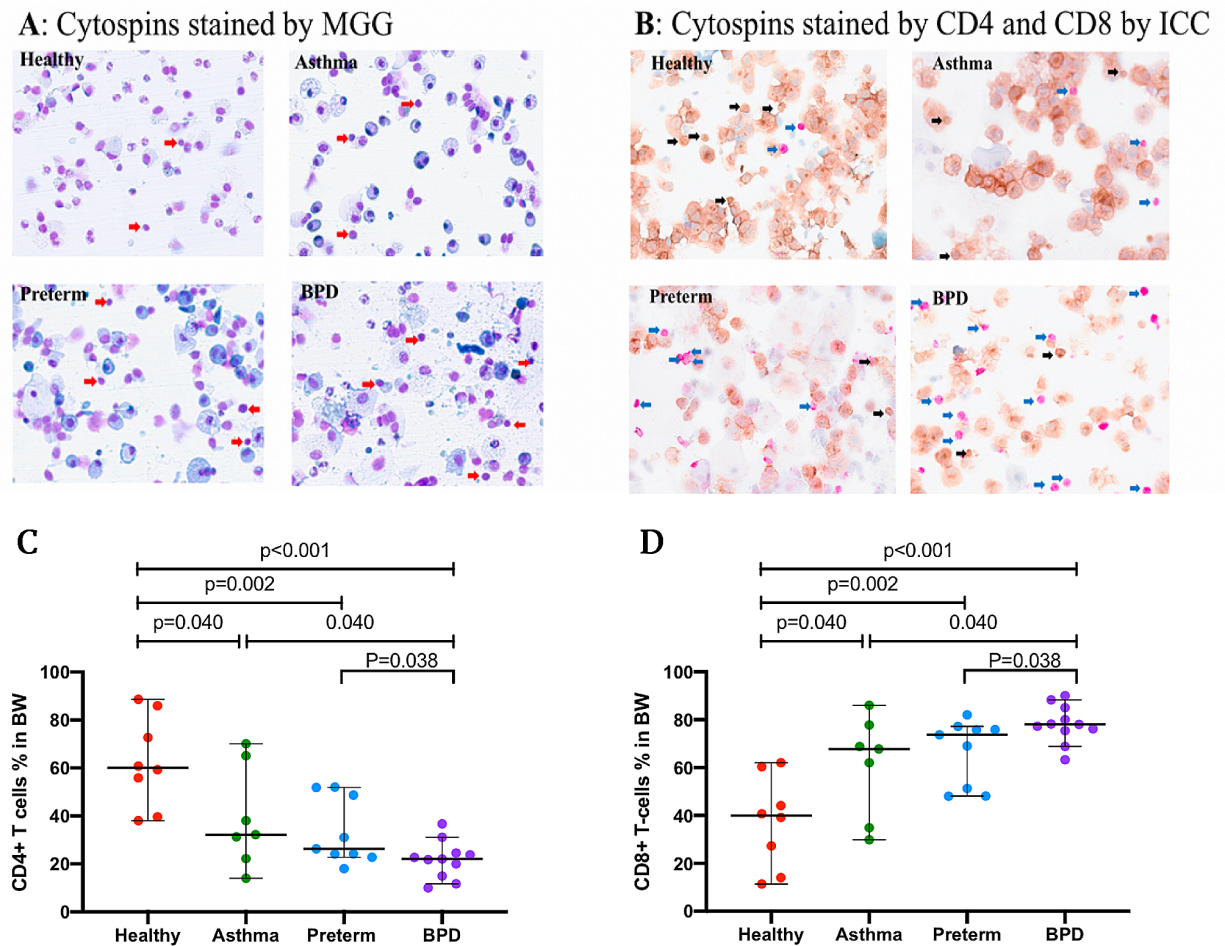
(**A**) Representative micrographs of cytospins from healthy, asthma, preterm, and BPD stained by MGG. Red arrows point to lymphocytes. (**B**) Cytospins stained for CD4 + and CD8 + T cells by ICC. Blue arrows point to the CD8 + T cells and black arrows points to the CD4 + T cells. (**C**) the proportion of CD4 + T cells of total cytotoxic T cells in BW, (**D**) proportion of CD8 + T cells of total cytotoxic T cells in BW (%). Abbreviations: BPD: bronchopulmonary dysplasia; MGG: May-Grünwald Giemsa, ICC: immunocytochemistry. BW: bronchial wash. Scale bar or magnification (BW cytospin x 400 magnification)

Bronchoscopy was performed according to a standardized protocol [33]. To collect cells from the large airways, we instilled 2 × 10 mL phosphate-buffered saline in two different segments in the right upper lobe which immediately was recovered by gentle suction. The recovered fluid was designated “bronchial wash” (BW). The BW fluid was quantified, debris and mucus were removed by filtration and the total number of cells was counted in a Burker chamber. A differential cell count in the bronchial wash was scored on cytospin slides, stained with May–Grünwald–Giemsa (MGG) and counted by examining 500 cells per subject  (Fig. 1, panel A). For comparison, the reported bronchoalveolar lavage data from the small airways was also collected [33].

### Double immunocytochemistry staining

As the number of cells was insufficient for flow cytometry analysis, the lymphocyte CD4 + and CD8 + T cells in the BW were analyzed using double immunocytochemistry (ICC) staining.

Slides from all subjects were counted, and only 35 subjects, with a total number of lymphocytes exceeding 50 cells per subject  (Fig. 1, panel B), were included in the final ICC analysis. The CD4 + and CD8 + T cells were identified by their stained color and cell morphology. Two different chromogens were used to label T cells (CD4 and CD8) in the BW cytospins: brown representing CD4 helper T cells and purple representing CD8 cytotoxic T cells. The prediluted monoclonal primary antibodies (Ventana, Roche) were as follows: clone SP35 for CD4 and clone C8/144B for CD8. The ICC was performed at the Department of Clinical Pathology and Cancer Diagnostics, Karolinska University Hospital, using the Benchmark Ultra Autostainer (Ventana, Roche) and protocols optimized for routine clinical samples.

### Correlation-based network analysis

To further elucidate factors correlating with large airway cells, we conducted correlation-based network analyses. This involved a comprehensive dataset that included perinatal and adult clinical data, lung function metrics and findings from both large and small airways. The approach allowed a detailed examination of the interconnections and relationships among these diverse data points. The analysis encompassed data from healthy, asthma, preterm, and BPD. Due to limited ICC data, all available information from this cohort (35 subjects) was included, while comprehensive non-ICC data from the total cohort (88 subjects) were included after omitting any missing entries. For further analysis, we have categorized premature subjects, including those with BPD and preterm conditions, into a single group. To analyze the relationships between large airway cells and other variables, we first used Spearman’s rank correlation, focusing on identifying significant correlation factors (association coefficient *r* > 0.3, *p*-value < 0.05). Next, we performed network analysis on pairs of factors, with edge widths representing the significance of the connections (-log10 (*p*-value)). We then grouped the factors using the leading eigenvector method in the Python package igraph [38]. The network analysis successfully categorized variables with strong associations into distinct clusters, with special focus on those intimately linked to large airway cells.

### Statistical analyses

The demographic data are presented as the median and range for continuous variables, unless stated otherwise. Categorical variables are presented as numbers and/or proportions. To compare continuous variables among the study groups, we used the Kruskal–Wallis test followed by the Mann–Whitney U-test, and the χ2 test was used for categorical variables. The analyses were conducted using R 4.1.1, Python 3.8, SPSS 28, and Prisma 8 environments.

## Results

### Participants

The LUNAPRE cohort consisted of 96 study subjects [34], of which 88 (18–23 years old, 46.6% male) underwent bronchoscopy with collection of bronchial wash, and therefore were included in the current study. The characteristics of the 88 adult study participants and their perinatal data are detailed in Table 1. The 43 preterm-born subjects (Preterm and BPD) had lower birth weight and gestational age compared to the 45 full-term-born subjects (Asthma and Healthy). Out of the 43 preterm-born subjects, 9 had mild BPD, 7 had moderate BPD, and 7 had severe BPD during the neonatal period. The median duration of CPAP and Supplemental O_2_ usage in all the preterm-born adults was 7 days and 26 days, respectively. Thirty out of 88 subjects were sensitized to common airborne allergens. Additionally, during the neonatal period, the mothers of 9 BPD subjects received corticosteroids. Unfortunately, the number of individuals who received postnatal systemic steroids is unknown. In the postnatal period, 15 BPD subjects used inhaled corticosteroids. Most important, all participants underwent a three-month wash-out period before their inclusion in the study (Table 1).

**Table 1:** Characteristics of study participants in adulthood and perinatal data

	Full term-birth		Preterm-birth	
	Healthy	Asthma	Preterm	BPD
**Adulthood**, n	23	22	20	23
Age	20.4 (19.1–20.8)	20.3 (19.5–21.9)	19.1 (18.9–19.4)*	19.6 (19.0-19.8)*
BMI	21.7 (20.5–23.6)	22.7 (20.6–25.7)	20.9 (19.4–23.7)	21.9 (19.4–24.1)
Gender (males)	11 (47.8)	9 (40.9)	10 (50.0)	11 (47.8)
Allergy (fur, flower, and food)^#^	0	20 (90.9)	2 (10.0)	7 (30.4)
Positive Phadiatop^®^	0	22(100)	6 (30.0)	2(8.7)
Methacholine test positive	0	22(100)	11 (55.0)	16 (69.7)
Post FEV_1_%, predicted	108.7 (101.2-112.5)	104.5 (94.1–110.0)	104.6 (98.6-115.9)	90.2 (83.8-100.7)*
Post FEV_1_ Z-score	0.75 (-0.04-1.08)	0.27 (-0.52-0.77)	0.40 (-0.12-1.32)	-0.85 (-1.37- -0.15)**
Post FVC %, predicted	105.1 (98.6-110.1)	103.0 (94.5-110.4)	98.1 (92.8-111.2)	94.9 (85.6–101.0)*
Post FVC Z-score	0.33 (-0.12- 0.69)	0.20 (-0.48-0.85)	-0.17 (-0.52-0.77)	-0.41 (-1.24-0.06)**
Post FEV_1_ /FVC	0.90 (0.86–0.92)	0.86 (0.85–0.89)*	0.92 (0.86–0.94)	0.83 (0.74–0.88)**
Post FEV_1_ /FVC Z-score	0.48 (-0.07-0.88)	-0.16 (-0.43-0.33)*	0.69 (0.01–1.20)	-0.79 (-1.79-0.03)**
FeNO, ppb	10.7 (9.7–16.5)	31.4 (11.6–49.2)*	13.1 (10.5–19.4)	14.7 (9.4–22.0)
DLCO (% pred)	87.0 (81.5–96.0)	81.5 (75.0–91.0)	78.5 (66.0-90.5)*	69.5 (65.0–77.0)***
RV/TLC	19.0 (17.0-22.5)	18.5 (14.0–23.0)	21.0 (18.0–23.0)	20.0 (17.0–24.0)
**Perinatal period**, n				
BPD severity, (mild, moderate, severe), n	N/A	N/A	N/A	9, 7, 7
Gestational age, weeks	40.1 (39.4–40.6)	39.9 (39.0–41.0)	30.1 (29.3–30.7)**	26.6 (26.3–28.2)**
Birth weight, gram	3457.5 (3245.0-3770.0)	3444.5 (3024.0-3935.0)	1470.0 (1132.5-1577.5)**	962.0 (782.5-1137.5)**
Apgar, 1 min	9 (8–9)	9 (9–9)	8 (7–9)	5 (4–8)**
Apgar, 5 min	10 (10–10)	10 (9–10)	9 (7–10)**	7 (7–9)**
Antenatal steroids	N/A	N/A	12 (60.0)	9 (39.1)
Instillation of surfactant,	N/A	N/A	2 (10.0)	8 (34.8)
Mechanical ventilation, days	N/A	N/A	0 (0–0)	6 (1–15)
CPAP, days	-N/A (delete-)	-N/A(delete-)	3.0 (2.0–5.0)	38.5 (30.0–52.0)
Supplemental O2, days	-N/A (delete-)	-N/A (delete-)	3.0 (1.0-8.5)	65.0 (58.0-83.5)
**Postnatal period**				
Inhaled corticosteroids	N/A	N/A	0	15 (65.0)

Note: Data are presented as median (IQR) or numbers (%). Abbreviations: BPD: bronchopulmonary dysplasia; BMI: body mass index; FEV_1_: forced expiratory volume in 1 s; FVC: forced vital capacity; DLCO: diffusing capacity of the lung for carbon monoxide; RV: residual volume; TLC: total lung capacity; FeNO: fractional exhaled nitric oxide; CPAP: continuous positive airway pressure; Apgar: Apgar-score; Post: post bronchodilator. ^#^: self-reported; (delete *:): N/A: not applicable; *: *P* < 0.05; **: *P* < 0.01; comparing BPD, preterm, and asthma group to healthy control group

### Lung function

Lung function, as previously reported [33, 34], showed that the BPD group had lower FEV_1_, FVC, FEV_1_/FVC, and DLCO values compared to healthy controls (Table 1). Of the preterm subjects, 55% and of the BPD subjects, 69.7% had a positive methacholine challenge test. According to the inclusion criteria in LUNAPRE [34], all asthma patients tested positive for methacholine, while none of the control group did. The asthma group had higher FeNO levels compared to the other three groups (Table 1).

### Differential cell counts in bronchial wash

The total cell yield in large airway BW samples ranged from 0.6 to 0.8 million cells, with no difference between the groups (Table 2). The most predominant cell type in all study groups was epithelial cells, accounting for 57.7% (median, IQR 49.1–69.7%) of the total cells.

**Table 2:** Cell counts in bronchial wash (BW) samples

	Full term-birth		Preterm-birth		*P*-value
	Healthy	Asthma	Preterm	BPD	
**Performed BW**	23	22	20	23	
Total cell yield (×10^6^)	0.78 (0.57–1.59)	0.61 (0.45–0.89)	0.60 (0.47–1.01)	0.59 (0.43–0.80)	0.150
Epithelial cells (%)	59.2 (50.2–75.0)	62.9 (45.4–71.6)	54.3 (47.7–68.6)	55.6 (50.2–61.4)	0.646
Macrophages (%)	17.0 (11.7–20.6)	23.6 (19.4–30.6)	26.4 (11.0-34.9)	25.8 (18.8–31.9)	0.094
Neutrophils (%)	12.8 (7.5–21.4)	9.9 (3.6–14.8)	11.3 (5.3–17.0)	12.8 (6.6–19.0)	0.303
Lymphocytes (%)	3.8 (2.5–4.7)	3.4 (3.2–5.2)	4.7 (4.0-7.5)	6.6 (5.1-8.0)**	< 0.001
Eosinophils (%)	0 (0-0.2)	0.3 (0–1)	0 (0–0)**	0 (0–0)**	< 0.001

Note: Data are presented as n or median (interquartile range). BPD: bronchopulmonary dysplasia. *:*P(delete p) p* < 0.05, **:*P(delete p)* *p* < 0.01, comparing BPD and preterm group to asthma group

**Fig. 2:**
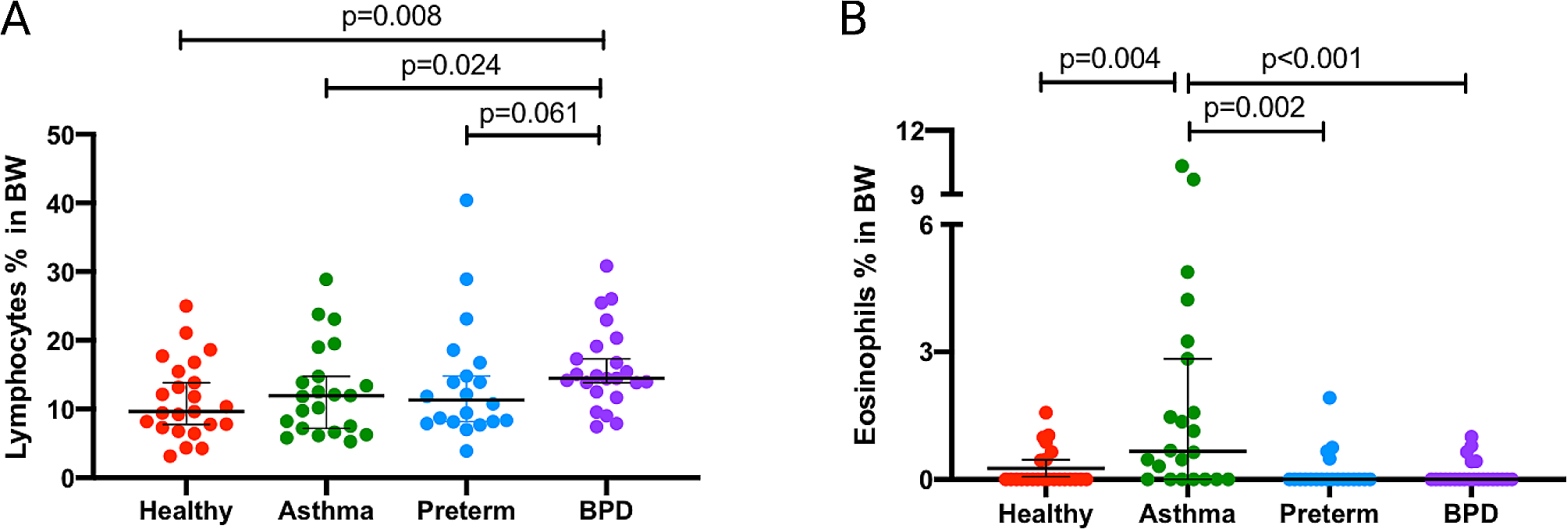
Percentages of lymphocytes (**A**) and eosinophils (**B**) in bronchial wash (BW) from healthy, asthma, preterm, and BPD. Epithelial cells were excluded. BPD: bronchopulmonary dysplasia; BW: bronchial wash

**Fig. 3:**
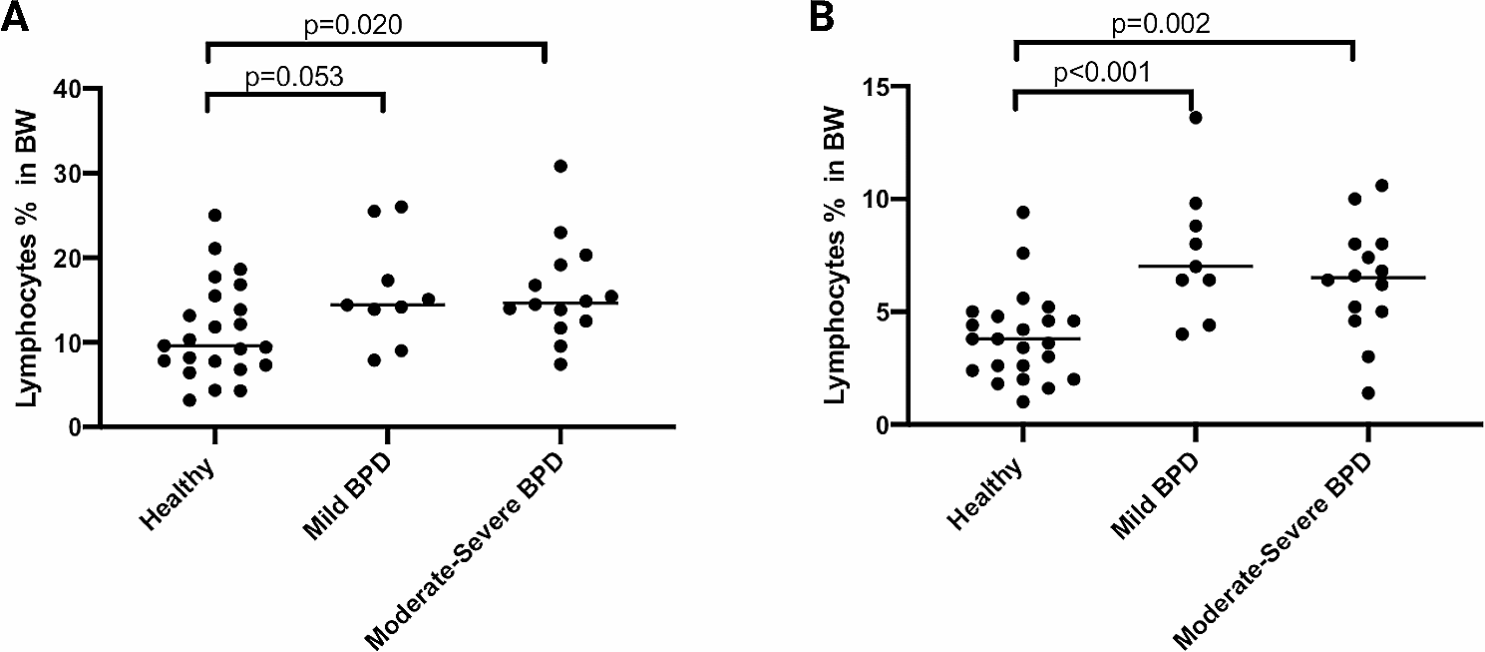
Lymphocyte counts from bronchial wash (BW) in BPD subjects divided in mild and moderate-severe disease compared to healthy. Panel (**A**) shows data with epithelial cells excluded. Panel (**B**) shows data with epithelial cells included. BPD: bronchopulmonary dysplasia; BW: bronchial wash

When excluding epithelial cells, the proportion of lymphocytes in the BW was significantly higher in the BPD group (median, 14.5%, Fig. 1, panel A) compared to healthy controls (9.6%, *p* = 0.008) and asthma (11.9%, *p* = 0.024). There was no significant difference in lymphocyte counts between the BPD and premature groups (11.3%) (*p* = 0.061). The proportion of eosinophils was higher in the asthma group compared to BPD (*p* = 0.001), preterm (*p* = 0.002), and healthy controls (*p* = 0.004, Fig. 2). Even after including epithelial cells in the analysis, the proportion of lymphocytes and eosinophils remained significantly different in BPD when compared to asthma (*p* = 0.002 and *p* < 0.001, respectively). The lymphocyte count in preterm participants was numerically higher than in healthy controls, although the difference was not statistically significant (*p* = 0.05) (Table 2). The proportion of lymphocytes was higher in mild and moderate-severe BPD, with 7.0% and 6.5%, respectively, compared to healthy controls (3.8%) (*p* < 0.001 and *p* = 0.002, respectively. Figure 3). However, there was no difference in lymphocyte counts between mild and moderate-severe BPD.

### Immunocytochemistry staining of BW lymphocytes

We performed immunocytochemistry (ICC) staining on CD4 + and CD8 + T-cells from cytospin samples that had more than 50 identifiable cells (*n* = 35). Supplementary Table 1 presents the characteristics of these subjects. We found no differences between the whole cohort and the ICC cohort, and the median gestational age did not differ. However, the percentage of CD4 + T-cells was significantly reduced in the BPD group compared to the preterm, asthma, and healthy control groups (*p* < 0.05 for all, Fig. 1, panels B and C). Additionally, the percentage of CD8 + T-cells was higher in the BPD group compared to the preterm (median, 73.7%, *p* = 0.038), asthma (67.8%, *p* = 0.040), and healthy (40.0%, *p* < 0.001 for all) groups (Fig. 1, panels B and D).

### Correlation between lymphocytes, CD8 + T cells and clinical characteristics

In all subjects, the proportion of CD8 + T cells correlated with FEV_1_-z-score in a negative manner (*p* = 0.007), and the proportion of lymphocytes displayed a trend towards correlation with FEV_1_-z-score (*p* = 0.065) (Table 3).Moreover, the proportion of lymphocytes and CD8 + T cells were both negatively correlated with gestational age, birth weight, and APGAR score at 1 min in all subjects (*r*=-0.436, *p* = 0.010; *r*=-0.452, *p* = 0.007; and *r*=-0.469, *p* = 0.008, respectively). In adults who were born prematurely (preterm and BPD combined), the proportion of lymphocytes was correlated with FVC-z-score in a negative manner (*r* = -0.324, *p* = 0.036), while the proportions of CD8 + T cells showed a correlation with FEV_1_-z-score in a negative manner (*r* = -0.448, *p* = 0.048). Additionally, the severity of BPD was correlated with CD8 + T cells in positive manner (*r* = 0.475, *p* = 0.034).

**Table 3:** Univariate correlation between correlation lymphocytes, CD8 + T cells and clinical characteristic

Variables	Lymphocytes		CD8 + T-cells		CD4 + T-cells	
	r	*P*-value	r	*P*-value	r	*P*-value
Gender (1 = male, 2 = female)	-0.244	0.022*	0.110	0.531	-0.110	0.531
BMI	-0.134	0.212	0.110	0.529	-0.110	0.529
APGAR, 1 min	-0.337	0.002**	-0.469	0.008**	0.469	0.008**
APGAR, 5 min	-0.227	0.043*	-0.574	0.007**	0.574	0.007**
Gestational age,	-0.348	< 0.001**	-0.436	0.010*	0.436	0.010*
BPD severity	0.240	0.121	0.475	0.034*	-0.475	0.034*
Birth weight, gram	-0.363	< 0.001**	-0.452	0.007**	0.452	0.007**
Post FEV1 Z-score	-0.199	0.065	-0.45	0.007**	0.45	0.007**
Post FVC Z-score	-0.213	0.048*	-0.281	0.102	0.281	0.102
Post FEV1/FVC Z-score	-0.037	0.735	-0.351	0.039*	0.351	0.039*
Post FEV1%, predicted	-0.187	0.083	-0.468	0.005**	0.468	0.005**
Post FVC %, predicted	-0.144	0.182	-0.290	0.091	0.290	0.091
Post_FEV1FVC	-0.094	0.386	-0.329	0.054	0.329	0.054
FeNO, ppb	0.244	0.022*	0.022	0.898	-0.022	0.898
DLCO (% pred)	-0.124	0.253	-0.527	0.001**	0.527	0.001**
RV/TLC	0.134	0.219	0.511	0.002**	-0.511	0.002**

Note: BPD: bronchopulmonary dysplasia; BMI: body mass index; FEV_1_: forced expiratory volume in 1 s; FVC: forced vital capacity; DLCO: diffusing capacity of the lung for carbon monoxide; RV: residual volume; TLC: total lung capacity; FeNO: fractional exhaled nitric oxide; APGAR: Apgar-score; Post: post bronchodilator; BMI: body mass index; APGAR: Apgar-score. *: *P* < 0.05; **: *P* < 0.01

In a multivariate regression analysis, after adjusting for GA days, birth weight, BMI, and Apgar scores, lymphocytes (HR: -0.281, 95% CI (-0.211- -0.002), *p* = 0.045) and BPD severity (-0.640, (-1.022- -0.293), *p* < 0.001) were found to be independently and negatively correlated with FEV_1_-z-score (Table 4).

**Table 4:** Multivariate regression analysis dependent as post- FEV_1_ z-scores

Independent variables	Standardized Coefficients Beta	95% CI		*P*-value
		lower	upper	
Lymphocytes	-0.281	-0.211	-0.002	0.045
BPD severity	-0.640	-1.022	-0.293	< 0.001
Gestational age	-0.047	-0.054	0.024	0.438
Birth weight	-0.153	-0.001	0.002	0.455
APGAR, 1 min	0.075	-0.178	0.294	0.619
APGAR, 5 min	-0.153	-0.288	0.216	0.77
BMI	0.075	-0.130	0.058	0.444

Note: BPD: bronchopulmonary dysplasia; BMI: Body mass index; FEV1: forced expiratory volume in 1 s; Post: post bronchodilator; APGAR: Apgar-score

### Correlation-based network analyses

**Fig. 4:**
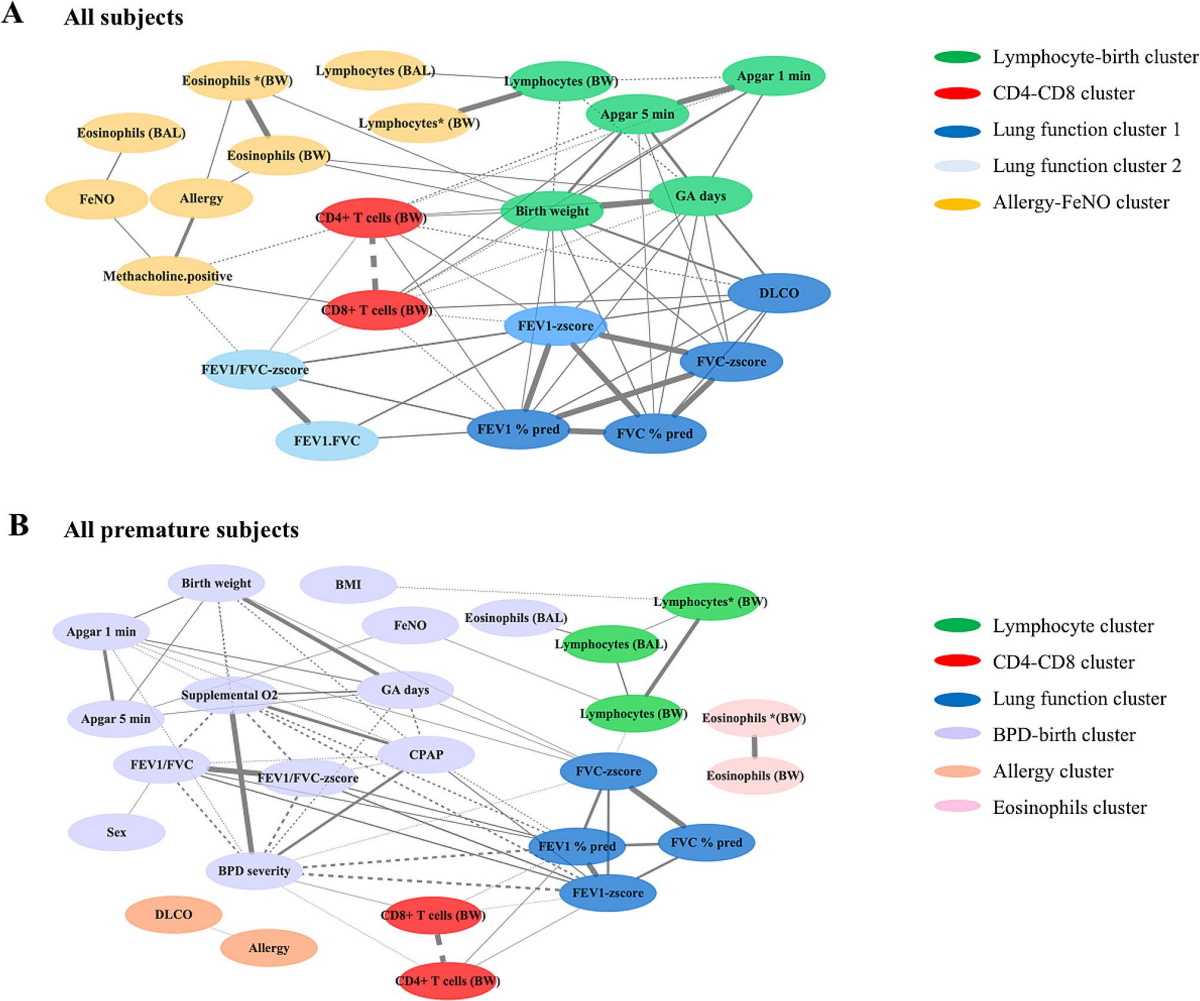
Correlation-based network analyses in (**A**) all subjects and in (**B**) all premature subjects (preterm + BPD). Significant pairs identified by the Spearman correlation test (coefficient *r* < 0.3, *p* < 0.05) are connected by edges with the width representing the significance (-log10(*p*-value)). Abbreviations: BPD: bronchopulmonary dysplasia; BMI: body mass index; FEV_1_: forced expiratory volume in 1 s; FVC: forced vital capacity; FeNO: fractional exhaled nitric oxide; CPAP: continuous positive airway pressure; APGAR: Apgar-score; Post: post bronchodilator; BW: bronchial wash; BAL: bronchoalveolar lavage; lymphocytes (BW) and *(BW): the proportion of lymphocytes included and excluded epithelial

In the network analysis, we incorporated data from the perinatal period, adulthood, and also BAL fluid data [33], to assess factors associated with lymphocytes and T cells in the large airways. Across all subjects (Fig. 4, panel A), four clusters were identified: Lymphocyte-Birth, CD4+ - CD8+, Lung Function, and Allergy-FeNO, linking lymphocytes with these specific variables. In the analysis focusing on premature subjects (Fig. 4, panel B), the network delineated clusters including Lymphocyte, CD4-CD8, Lung Function, BPD-Birth, Allergy, and Eosinophils. This highlights the connections between lung development and immune responses, with the lung function and BPD-birth clusters specifically associated with lymphocytes and CD4 + and CD8 + T cells. Additionally, two distinct clusters related to allergies and eosinophils were identified in this subgroup (Fig. 4, panel B). However, these clusters did not exhibit a direct link to lymphocytes or T cells. Both panels showed correlations between lymphocytes and lung development, with additional focus in premature subjects on BPD outcomes and distinct immune responses (Fig. 4).

## Discussion

We demonstrate an increase in the number and proportions of lymphocytes and CD8 + T-cells in the large airways, and a decrease in the proportions of CD4 + T-cells in adults with a history of BPD compared to healthy control subjects and patients with asthma. We found a negative correlation between cytotoxic CD8 + T cells in the large airways and lung function. Furthermore, lymphocyte proportion was identified as an independent factor negatively associated with airway obstruction in adults born prematurely. These findings suggest a potential involvement of these cells in the mechanisms leading to the development of chronic airway disease as a result of preterm birth.

In this single-center study involving bronchoscopy, the LUNAPRE cohort is substantial, comprising 88 individuals, providing comprehensive contextual and baseline data. In our previous study [33], we demonstrated that adults with a history of BPD exhibit an increased proportion of activated CD3 + CD8 + cells, a decrease in CD3 + CD4 + T cells, and a reduced CD4/CD8 ratio in BAL fluid from the small airways. Since obstructive airway disease can also affect the large airways, our current research focuses on the cellular profile in the large airways, particularly in adults with a history of BPD, with the aim of enhancing our understanding of the role of lymphocytes and T cells in chronic airway diseases. To achieve our research objectives, we conducted bronchial washes and cytospin analysis, with a specific emphasis on CD4 + and CD8 + T cells in this subgroup. Due to limited cell recovery, we employed immunocytochemistry instead of flow cytometry. Only samples containing a minimum of 50 lymphocytes per slide, as assessed by expert pathologists, were included in the assessment of CD4 + and CD8 + T cells, resulting in a final analysis of 35 subjects.

Our study unveils the disparities between asthma and BPD. Asthma is primarily associated with CD4(+) T cells [39, 40]. Conversely, corticosteroid-resistant pathways involving neutrophils and CD8 + T cells may involve common triggers of asthma exacerbations [26]. Significantly, we observed a strong correlation between CD8 + T cells and airway obstruction in individuals with a history of BPD and preterm subjects. This finding suggests that the activation of CD8 + T cells in former BPD patients may contribute to tissue remodeling and subsequent declines in lung function [41]. Furthermore, it’s worth noting that lymphocyte proportions increase in adults with a history of BPD, but we did not find significant differences in the counts of other cell types (neutrophils, macrophages, and epithelial cells) among the various groups. In a prior study, Galderisi et al. [32] reported elevated lymphocyte levels, particularly CD8 + T cells, in bronchial biopsies from three patients (aged 10–15 years) with severe BPD, as confirmed by immunohistochemistry. This discovery provides additional support for our hypothesis regarding the involvement of these cells in the disease mechanisms within the large airways.

In addition, individuals with a history of BPD frequently exhibit asthma-like symptoms, that may lead to prescription of asthma medications including inhaled corticosteroids. This practice, however, may not be congruent with the pathophysiology of BPD. Our findings of increased cytotoxic T cells in the airways resembles what is previously reported in COPD [42], which is a disease predominantly treated with bronchodilators. This underscores the necessity for precise diagnostic and management strategies and individualized management in daily clinical practice. Concurrently, recent guidelines and research advocate for bespoke management of BPD and related chronic neonatal lung diseases. The European Respiratory Society’s guidelines, for instance, propose specific monitoring and treatment modalities, including the conditional application of bronchodilators for asthma-like symptoms and discouraging the routine use of inhaled or systemic corticosteroids [43]. Hence, in managing respiratory symptoms in adults with the history of BPD, the customary employment of inhaled corticosteroids warrants circumspection.

In our study, we used correlation network analysis to explore potential factors in disease development by revealing connections between disease-related clusters [44–46]. Analyzing data from both adult and perinatal stages, we uncovered interactions between lymphocytes, T cells, perinatal conditions, and adult lung function, deepening our understanding of disease dynamics. Our network analysis identified distinct clusters, including lymphocyte-birth, CD4+ - CD8+, lung function, and allergy-FeNO, highlighting complex relationships between lymphocyte profiles and clinical characteristics. In premature subjects, the lung function and BPD-birth clusters underscore a significant link between early lung development and immune cell profiles. Additionally, our analyses revealed connections between lymphocytes measured in bronchial wash and BAL, indicating consistent immune patterns across different respiratory compartments. This approach elucidated essential intercorrelations between clusters, preserving data complexity and emphasizing key patterns and relationships [44, 45].

Given the limited existing literature on the association between airway inflammation profiles in prematurely born adults and their lung function, we sought to explore the impact of BPD severity. We found that both mild and moderate-severe BPD subjects had higher lymphocyte proportions than healthy counterparts, suggesting lymphocytic inflammation may begin during the mild BPD stage. However, small sample sizes in the mild and moderate-severe BPD subgroups limit clear distinctions in our findings. Nevertheless, our study highlights BPD’s potential heterogeneity, stressing the need to identify intervention targets for mitigating COPD risk in this population.

LUNAPRE is a cohort of prematurely born adults that underwent extensive characterization, including full airway sampling, lung function testing, clinical phenotyping, and comprehensive data on neonatal conditions and demographics. We utilized airway sampling to examine the inflammatory profiles in the lungs associated with BPD. We included subjects, born between 1992 and 1998, from a period when the transition to modern neonatal care occurred. In our study, only a few participants received surfactant and prenatal corticosteroids. Recent research [47] highlights the benefits of noninvasive respiratory support for reducing lung injury and BPD incidence in preterm infants. Despite advances in neonatology improving survival rates, the incidence of BPD among survivors remains high [48, 49]. A review [50] on adult survivors of BPD emphasizes the shift from the “old” form of the disease, linked to perinatal mechanical ventilation, to a “new” form due to earlier lung development interruption. This underscores the varied manifestations of BPD in adulthood, including asthma-like disease and pulmonary hypertension, necessitating the transition from pediatric to adult pulmonary care. Additionally, former BPD patients are often managed as asthmatics [34, 51], and our study benefits from age-matched control groups of healthy individuals and asthmatics.

Nonetheless, we acknowledge some limitations. Our sample size, while one of the largest BPD cohorts examined with bronchoscopy, is relatively small. Additionally, the absence of surfactant and prenatal corticosteroids during the study may have influenced outcomes. Furthermore, our research primarily focused on preterm infants with a median gestational age of 26.6 weeks, potentially not fully representative of those born even earlier under contemporary neonatal care standards. Future research should target contemporary adult cohorts with a history of preterm birth to enrich our understanding of BPD’s current status and evolution, crucial for updating neonatal care protocols.

In conclusion, this study presents the first investigation of the inflammatory profile in the large airway of young adults born preterm, including those with a history of BPD. We report key findings showing an enhanced proportion of lymphocytes and CD8 + T-cells in the large airway of adults with a history of BPD, which were negatively correlated with airway obstruction. The correlations between cytotoxic T cells and lung function in preterm subjects suggest that T-cells may be involved in the mechanisms underlying chronic airway obstruction in adults with a history of BPD. Our findings indicate the involvement of adaptive and innate immune responses and alterations in inflammatory profiles in the development of airway disease due to preterm birth.

### Electronic supplementary material

Below is the link to the electronic supplementary material.

Supplementary Material 1

## Data Availability

Anonymized data from the current study is available on reasonable request.

## Declarations

### Additional information

Abstracts of data included in the paper have previously been presented at the European Respiratory Society (ERS), Paris 2018 and at the Swedish Respiratory Society Annual Congress, Stockholm 2019.

## Abbreviations

BAL: Bronchoalveolar lavage
BMI: Body mass index
BPD: Bronchopulmonary dysplasia
BW: Bronchial wash
CMM: Center for Molecular Medicine
COPD: Chronic obstructive lung disease
CPAP: Continuous positive airway pressure
DLCO: Diffusing capacity of the lung for Carbon monoxide
FeNO: Fractional exhaled nitric oxide
FEV1: Forced expiratory volume in 1 s
FVC: Forced vital capacity
GA: Gestational age
GIAR: Gunma University Initiative for Advanced Research
IQR: Inter quartile range
ICC: Immunocytochemistry
INF: Interferon
LUNAPRE: Lung Obstruction in Adulthood of Prematurely Born study
MGG: May–Grünwald–Giemsa
N/A: Not applicable
Post: post bronchodilator
PMA: Postmenstrual age
RV: Residual volume
TLC: Total lung capacity
VC: Vital capacity
