# Supplementary material for: Large airway T cells in adults with former bronchopulmonary dysplasia
Source: Respir Res. 2024 Feb 9;25:86. doi: 10.1186/s12931-024-02717-1 (PMC10858477; doi:10.1186/s12931-024-02717-1)
Supplement: Supplementary file 1 — Supplementary Material 1 [file 12931_2024_2717_MOESM1_ESM.docx]

**Supplement table 1: Characteristics of study participants in adulthood and perinatal data in ICC cohort**

|  | **Full term-birth** |  | **Preterm-birth** | |
| --- | --- | --- | --- | --- |
|  | **Healthy** | **Asthma** | **Preterm** | **BPD** |
| **Adulthood,** n | 8 | 7 | 9 | 11 |
| Age | 20.8 (19.7-21.3) | 19.8 (19.4-20.6) | 19.1 (18.9-19.4) | 19.2 (18.7-19.6) |
| BMI | 22.3 (21.2-23.3) | 23.9 (22.1-26.3) | 20.4 (18.1-23.4) | 22.1 (19.8-25.1) |
| Gender (males, n (%)) | 3 (37.5) | 3 (42.9) | 2 (22.2) | 2 (18.7) |
| Allergy (fur, flower, and food)^#^, n (%) | 0 | 6 (85.7) | 1 (11.1) | 5 (45.5) |
| Positive Phadiatop^®^ | 0 | 7(100) | 3 (33.3) | 1(9.1) |
| Post FEV_1_ %, predicted | 105.6 (99.5-113.3) | 94.1 (87.0-110.4) | 103.6 (102.3-115.7) | 85.0 (83.9-98.2)** |
| Post FEV_1_ Z-score | 0.49 (-0.04-1.15) | -0.52 (-1.10-0.91) | 0.31 (0.20-1.27) | -1.31 (-1.37-0.26)** |
| Post FVC %, predicted | 102.6 (93.1-106.8) | 94.5 (91.6-107.5) | 98.0 (96.2-108.3) | 96.4 (92.0-100.9) |
| Post FVC Z-score | 0.22 (-0.56-0.54) | -0.48 (-0.71-0.63) | -0.16 (-0.31-0.72) | -0.29 (-0.65-0.004) |
| Post FEV_1_ /FVC | 0.91 (0.88-0.94) | 0.86 (0.85-0.87)* | 0.92 (0.89-0.93) | 0.85 (0.74-087)* |
| Post FEV_1_ /FVC Z-score | 0.61 (0.17-0.98) | -0.20 (-0.40-0.05)* | 0.57 (0.13-1.33) | -0.53 (-1.98- -0.14)* |
| FeNO, ppb | 13.6 (10.6-20.9) | 30.0 (19.4-41.1) | 13.1 (11.0-15.0) | 14.7 (11.0-29.0) |
| **Perinatal period**, n |  |  |  |  |
| BPD severity, (mild, moderate, severe), n | N/A | N/A | 0 | 2,5,4 |
| Gestational age, weeks | 40.0 (39.5-40.1) | 40.0 (38.9-41.1) | 30.1 (29.0-30.7)** | 26.6 (25.9-28.7)** |
| Birth weight, gram | 3400.0 (3245.0-3662.5) | 3384.0 (3289.5-3605.0) | 1425.0 (1090.0-1600.0)** | 940.0 (679.0-1082.5)** |
| Apgar, 1 minute | 9.0 (8.5-9.0) | 9.0 (7.5-9.0) | 8.0 (6.5-9.0) | 6.0 (3.0-8.5) |
| Apgar, 5 minutes | 10.0 (9.5-10.0) | 10.0 (8.5-10.0) | 8.0 (6.0-9.5) | 8.0 (6.5-9.0)* |
| Antenatal steroids, therapy | N/A | N/A | 6 (66.7) | 4 (36.4) |
| Instillation of surfactant | N/A | N/A | 1 (11.1) | 5 (45.5) |
| Mechanical ventilation, days | N/A | N/A | 0 (0-0) | 6 (1-15) |
| CPAP, days | N/A | N/A | 2.0 (2.0-3.0) | 35.0 (30.5-45.5) |
| Supplemental oxygen, days | N/A | N/A | 3.0 (1.0-9.0) | 72.0 (63.0-93.0) |
| **Postnatal period** |  |  |  |  |
| Inhaled corticosteroids | N/A | N/A | 0 | 8 (72.7) |

Note: Data are presented as median (IQR) or numbers (%). Abbreviations: ICC: immunocytochemistry; BMI: body mass index; FEV_1_: forced expiratory volume in 1 s; FVC: forced vital capacity; FeNO: fractional exhaled nitric oxide; BPD: bronchopulmonary dysplasia; Apgar: Apgar-score; CPAP: continuous positive airway pressure; N/A: not applicable. *: P < 0.05; **: P < 0.01; comparing BPD, preterm, and asthma group to healthy control group.
